# LGBTQ+ Veterans Report Greater Subjective Cognitive Impairment and Risk Factors for Alzheimer's Disease and Related Dementias

**DOI:** 10.1002/alz70860_106688

**Published:** 2025-12-23

**Authors:** Jace D. Flatt, Joel G Anderson, Brittany Klenczar, Ethan C. Cicero, Michelle M Hilgeman, Jennifer Pharr, Jalal Uddin

**Affiliations:** ^1^ University of Nevada Las Vegas School of Public Health, Las Vegas, NV, USA; ^2^ College of Nursing, University of Tennessee, Knoxville, TN, USA; ^3^ Emory University, Atlanta, GA, USA; ^4^ Veterans Affairs, Tuscaloosa, AL, USA

## Abstract

**Background:**

Alzheimer's disease and related dementias (ADRD) among the lesbian, gay, bisexual, transgender, queer, and/or additional marginalized sexual/gender identities (LGBTQ+) Veteran population is understudied. Research has found that LGBTQ+ Veterans experience several risk factors for ADRD, including vascular diseases and mental health conditions. Self‐reported memory problems or subjective cognitive impairment (SCI) may be an early marker of ADRD risk.

**Method:**

We examined data from 35 U.S. states and two U.S. territories from the 2023 Behavioral Risk Factor Surveillance System (BRFSS) that included SCI, Veteran status, and the optional sexual orientation and gender identity (SOGI) module. BRFSS is an annual cross‐sectional survey that uses random digit dialing via cellphones and landlines to assess the health and health behaviors of individuals aged 18+ residing in the U.S. The sample included adults who identified as a Veteran (*n* = 27,009) and were aged 45+ (mean: 69.1 years, range: 45 to 80+). SCI was based on reporting serious difficulty concentrating, remembering, or making decisions. Multivariable logistic regression was used to examine associations between SCI and LGBTQ+ identity adjusting for demographic characteristics (age, race/ethnicity, education and income).

**Result:**

Nearly 4% of the sample identified as LGBTQ+ (*n* = 1039). Compared with non‐LGBTQ+ Veterans, LGBTQ+ Veterans were significantly younger (mean 67.8 vs. 69.2 years) and more likely to report being unable to work (8.6% vs. 4.6%), have an annual income of ≤$25,000 (15.0% vs. 9.2%), live alone (33.1% vs. 27.4%), and have poor/fair health (27.4% vs. 23.0%). In terms of ADRD risk factors, LGBTQ+ Veterans were more likely to report having a depressive disorder (27.7% vs. 15.7%) and functional impairments (10.0% vs. 7.7%) compared with non‐LGBTQ+ Veterans. Over 17% of LGBTQ+ Veterans reported SCI compared with 11% of non‐LGBTQ+ Veterans (*p* <0.001). After accounting for demographic characteristics, LGBTQ+ Veterans were 62% more likely to report SCI (adjusted OR:1.62; 95% CI:1.30, 2.01) than non‐LGBTQ+ Veterans

**Conclusion:**

This study highlights greater concern for SCI among LGBTQ+ Veterans compared with non‐LGBTQ+ Veterans. There is a critical need for better understanding risk and protective factors associated with ADRD among LGBTQ+ Veterans and the needs, challenges, and health experiences of LGBTQ+ Veterans living with ADRD.

